# A pragmatic pilot phase II randomised controlled trial of prothrombin complex concentrates (PCC) versus fresh frozen plasma (FFP) in adult patients who are undergoing heart surgery (PROPHESY)

**DOI:** 10.1186/s13063-019-3759-8

**Published:** 2019-12-09

**Authors:** Laura Green, Neil Roberts, Jackie Cooper, Jane Field, Ravi Gill, Andrew Klein, Seema Agarwal, Simon Stanworth, Atholl Johnston, Vivienne Monk, Ben O’Brien

**Affiliations:** 1 Blizard Institute, Queen Mary University of London, NHS Blood and Transplant and Barts Health NHS Trust, 4 Newark Street, Whitechapel, London, E1 2AT UK; 2 St Bartholomew’s Hospital, West Smithfield, London, EC1A 7BE UK; 3 Barts Cardiovascular Clinical Trials Unit (CVCTU), William Harvey Research Institute, Heart Centre, Barts and The London School of Medicine, Queen Mary University of London, Charterhouse Square, London, EC1M 6BQ UK; 4 Southampton General Hospital, Tremona Road, Southampton, SO16 6YD UK; 5 Papworth Hospitals NHS Foundation Trust, Papworth Everard, Cambridge, CB23 3RE UK; 6 Manchester Royal Infirmary, Oxford Road, Manchester, M13 9WL UK; 7 Oxford University Hospitals NHS Foundation Trust, Headley Way, Oxford, OX3 9BQ UK; 8 William Harvey Research Institute, Heart Centre, Barts and The London School of Medicine, Queen Mary University of London, Charterhouse Square, London, EC1M 6BQ UK; 9 Outcomes Research Consortium, Cleveland Clinic, Cleveland, OH USA

**Keywords:** Cardiac surgery, Bleeding, Fresh frozen plasma, Prothrombin complex concentrate, Randomised controlled trial, Pilot study

## Abstract

**Background:**

Fresh frozen plasma (FFP) is the accepted standard treatment for clotting factor replacement in bleeding patients during or immediately after cardiac surgery. In the United Kingdom prothrombin complex concentrate (PCC) is not licensed in this setting, although it is being used in Europe because it has a higher concentration of clotting factor levels, and it can be administered rapidly and in small volume, resulting in less volume overload during cardiac surgery.

**Methods:**

PROPHESY is a pragmatic, single-centre, open-label, randomised, controlled pilot trial that will assess whether it is feasible to perform a large trial in the future that will compare PCC versus FFP in patients who are bleeding (not on warfarin) and who require blood transfusion. Over a 15-month period, 50 patients will be randomised to PCC versus FFP if they develop active bleeding within 24 h of cardiac surgery and for whom the clinician has decided to administer FFP for treatment of bleeding. Standard laboratory and point-of-care assessments will be performed as per routine practice, and additional research blood samples will be taken at three time points to assess haemostasis. Subjects will be assessed daily up to hospital discharge or 30 days or death (whichever occurs first) and will be seen in follow-up for 90 days after surgery to assess for thromboembolic complications and hospital re-admission since discharge. Quality-of-life assessment will be performed pre-surgery and at 90 days post-surgery. We will also perform qualitative research with clinical experts and patients to explore the understanding of and experience with the interventions, as well as adherence to study procedures and protocol.

**Discussion:**

There have been no randomised controlled trials that have compared the safety and efficacy of FFP versus PCC in cardiac surgery patients who are bleeding. This pilot study will assess if individual components of a large trial are deliverable to assess the safety and efficacy of the two blood products in the future.

**Trial registration:**

EudraCT, 2018-003041-41; ClinicalTrials.gov, NCT03715348. Registered on 29 July 2018.

## Background

Approximately 30,000 cardiac procedures are performed each year in the United Kingdom, and it is estimated that approximately 10% of all blood supplied by the National Blood Service is used during these procedures. Bleeding after cardiac surgery that requires blood transfusion is associated with significant morbidity and mortality, resulting in substantial costs to healthcare systems [1]. The national comparative audit in the United Kingdom in 2011, which incorporated data from 66% of all UK cardiac centres, showed that the overall blood transfusion rate was high across all procedures, with fresh frozen plasma (FFP) being administered in over 20% of patients undergoing valve replacement or repair surgeries and in 30% of patients undergoing combined coronary artery bypass graft + valve repair/replacement surgeries [2].

FFP is the accepted standard treatment for replacement of clotting factors in bleeding patients undergoing cardiac surgery; yet, in a recent Cochrane review only 1 study out of 14 trials (*n* = 738 participants) identified has evaluated the efficacy of FFP in bleeding patients, and this was underpowered to determine outcomes in mortality [3]. Taking into consideration that blood transfusion is not without risks, other haemostatic agents, such as prothrombin complex concentrate (PCC), are being explored by clinicians for management of bleeding, including in the peri-operative phase for patients undergoing cardiac surgery. Potential advantages of PCC over FFP include increased concentration of clotting factors leading to faster and more sustained reversal of coagulopathy, improved ease and speed of administration, reduced fluid volume (20–40 ml compared with up to 1000 ml with FFP), and reduced incidence of immunomodulatory side effects.

However, to date there have been no randomised controlled trials (RCT) that have compared the clinical efficacy and safety of PCC versus FFP in bleeding cardiac surgery patients who are not taking vitamin K antagonists (e.g., warfarin), and this was highlighted in a recent systematic review [4]. Several observational studies have demonstrated that PCC is safe in this setting and that its administration is associated with reduced blood transfusion requirements, albeit with no difference in other outcomes [5, 6]. However, clinical equipoise and the lack of high-quality evidence means that an RCT is required to determine how PCC compares with FFP. Prior to such a trial, a pilot study is required to determine if a large-scale RCT is possible, and this is the hypothesis of our single-centre RCT.

## Methods

### Study design

**Fig. 1:**
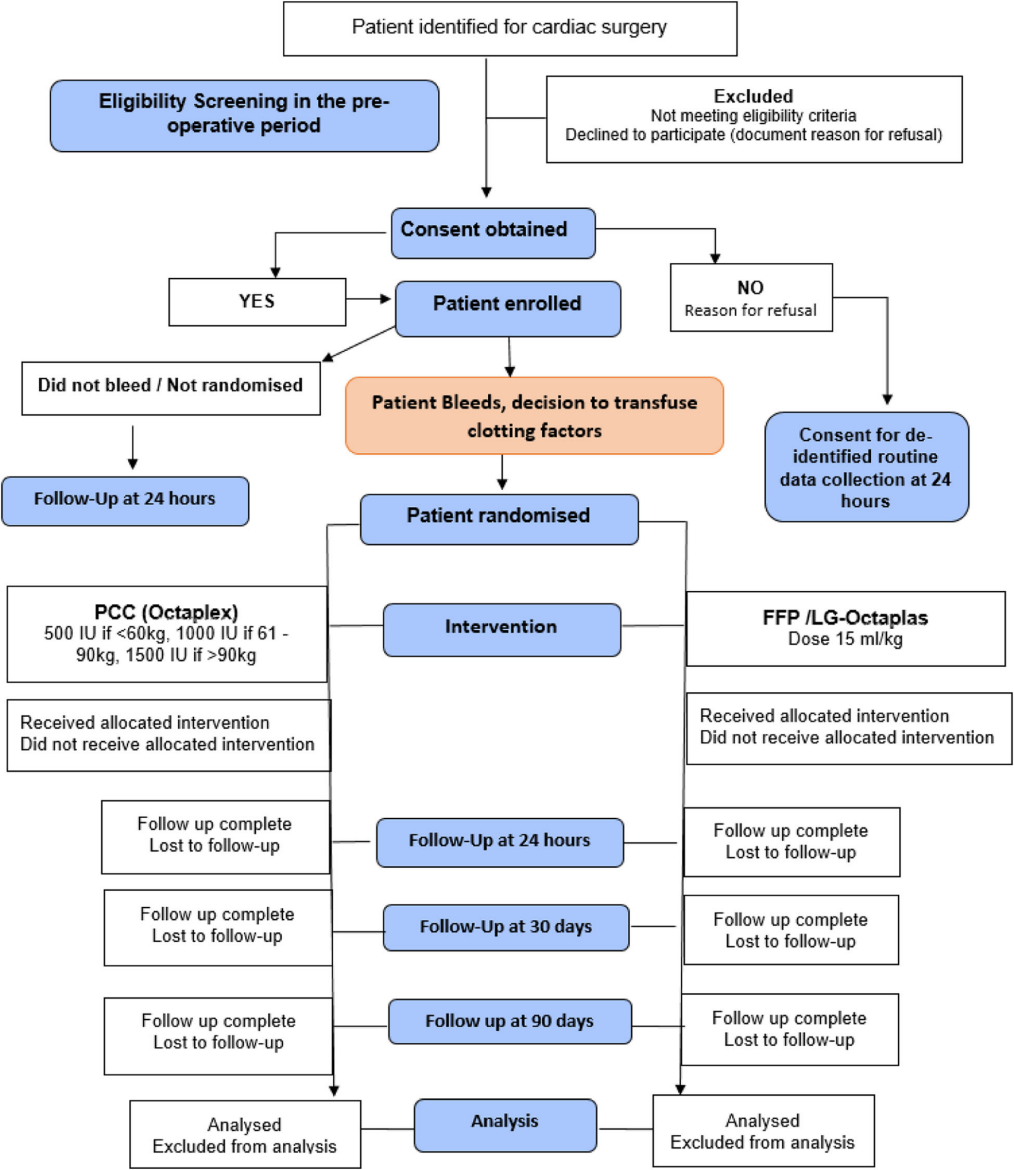
Study flowchart

The study design is a single-centre (Barts Health NHS Trust), open-label, non-blinded, pragmatic, pilot RCT (see Fig. 1 for study flowchart).

### Aim and objectives

The aim of this study is to determine if it is feasible to deliver a large trial in the future that will compare FFP versus PCC in cardiac surgery patients who are bleeding within 24 h of surgery.

#### Primary objective

The primary objective is to evaluate the recruitment rate, defined as the proportion of subjects who consent to the study (of all those eligible) and receive the intervention.

#### Secondary objectives

The following are secondary objectives of this study:

1. Assess the delivery of different components of the trial, protocol compliance and violation, and the ability to collect outcome data
2. Compare the impact of FFP and PCC on the haemostatic capacity of bleeding patients through the use of standard clotting tests and other global clotting tests
3. Obtain input from patients, members of the public and healthcare professionals on the design/running of the large trial, as well as identify the most important primary/secondary outcomes for the larger trial

#### Primary outcome

The primary outcome will be the proportion of participants who receive the intervention within 24 h of surgery, out of all eligible participants.

#### Secondary endpoints

The secondary endpoints of the study are as follows:

- Time to administration of study drug (PCC) or control (FFP) to patient, defined as time in minutes from telephoning laboratory to first administration to patient
- Proportion of patients for whom clinical outcome data were collected up to 90 days, or death, whichever occur first
- Proportion of patients who consent and are randomised within 24 h of surgery
- Proportion of patients who consent and are not randomised within 24 h of surgery
- Proportion of patients for whom timing of administration and completion of intervention(s) are documented
- Proportion of patients in whom there is protocol adherence and protocol violation
- Proportion of patients who do not consent to intervention but agree to consenting of their de-identified data for up to 24 h after surgery
- Obtain data on event rates in both groups to help estimate the sample size for the large trial. Event rates will be assessed at 24 h and 7, 14, 21 and 30 days, or upon discharge or death, whichever is first. Clinical outcomes assessed at these time points include those described under ‘Study assessment’, such as total days in intensive care unit, any organ failure, thrombosis, acute transfusion reaction, infections, duration of organ support and mortality.

### Study population

A total of 50 patients will be randomised over a 15-month period, with follow-up at 90 days or death, whichever occurs first. Consent will be obtained from all patients prior to participation in the trial.

#### Inclusion criteria

The study will include adult patients (aged > 18 years) who are able to give consent and undergoing elective or non-elective cardiac surgery, excluding procedures listed below under ‘Exclusion criteria’.

#### Exclusion criteria

Exclusion criteria are inability to consent; patients refusing blood transfusion for any reason; first time isolated coronary artery bypass graft; first time isolated aortic valve replacement (excluding active endocarditis); thoraco-abdominal surgeries; minor surgeries that do not involve cardiopulmonary bypass; use of warfarin within 4 days; use of direct oral anticoagulants (i.e., dabigatran, rivaroxaban, apixaban or edoxaban) within 48 h or 72 h, depending on estimated glomerular filtration rate; inherited bleeding disorder; pregnancy; known or suspected allergy to FFP, octaplasLG (Octapharma, Lachen, Switzerland) or PCC; known or suspected allergy to heparin, sodium citrate dihydrate, sodium dihydrogen phosphate dihydrate and glycine; history of heparin-induced thrombocytopenia; immunoglobulin A (IgA) deficiency with known antibodies against IgA; documented venous thromboembolism in the last 3 months; documented antiphospholipid syndrome; severe protein S deficiency; and participation in another clinical trial where the patient has received an investigational medicinal product in the last 3 months.

For women of childbearing age (< 50 years old) a urine pregnancy test will be performed for eligibility purposes. There will be no other study-specific screening procedures.

To determine the bleeding rate, routine clinical data will also be collected for up to 24 h for (1) eligible participants who have consented to take part in the study but are not randomised because they did not develop bleeding and (2) eligible participants who have not consented to take part in the main study but have consented to the collection of de-identified routine data.

### Randomisation process

The pragmatic nature of the study means that the decision whether to administer the intervention will be based on clinicians’ judgement, so that when a patient is actively bleeding within 24 h of surgery and a clinician has decided that FFP is needed to treat the bleeding, the patient will be randomised by the transfusion laboratory to either a single dose of FFP (fresh frozen plasma or octaplasLG) or 4-factor PCC (octaplex; Octapharma) using a web-based electronic database. Block randomisation will be used to ensure balance of treatments. The algorithm will be written by the study statistician using the ralloc command in Stata software (StataCorp, College Station, TX, USA), and a randomisation list will be produced. In the United Kingdom it is recommended that, as a variant Creutzfeldt-Jakob disease risk reduction measure, individuals born after 1 January 1996 should be transfused non-UK plasma, and this has been the practice since 1999 [7]. At the study site, octaplasLG is the standard of care for management of such patients who are bleeding. Doses of intervention will be calculated according to subject weight and as per the dosing schedules below.

**Table:** 

Subject weight	FFP or octaplasLG
≤ 60 kg	3 units
61–90 kg	4 units
> 90 kg	5 units
Subject weight	octaplex (IU)
≤ 60 kg	500 (one vial)
61–90 kg	1000 (two vials)
> 90 kg	1500 (three vials)

If the subject continues to bleed after this first single dose of study treatment, standard care for the treatment of bleeding will continue as per hospital protocol, and this may include having additional FFP. However, no further PCC will be administered to these subjects.

#### Study assessments

Subjects will have laboratory assessments with standard routine care tests and thromboelastography. Research blood samples will also be taken at three time points (pre-intervention and 1 h and 24 h post-intervention) to perform a more detailed analysis of haemostatic capacity of subjects (see Table 1 in Appendix, Additional file 1). Samples will be stored at − 70 °C ± 10 °C within 4 h of collection until they have been analysed, and no longer than 3 years after their collection. After analysis all samples will be destroyed. Samples will also be destroyed if the participant withdraws consent.

**Table 1:** Assessments of randomised subjects

Study Procedure	Screening pre-operatively	Prior to surgery (time 0)	Prior to randomisation – intervention	1 h post-study intervention	24 h post-surgery	Days 7, 14, 21	Day 30 (discharge/death)	Day 90 (discharge/death)
Visit windows	− 14 days	Day 0	Day 0	Day 0	Day 1	+/− 1 day	+/− 2 days	+/− 7 days
Screening - assess eligibility (includes urine pregnancy test)	X							
Informed consent	X							
Patient characteristics	X							
Assessment by surgical team	X							
Assessment by anaesthesiologist	X							
Blood tests^a^ – FBC	X		X	X	X			
Blood tests^a^ - group and screen samples	X	X						
Blood tests^a^ – liver and renal function tests	X				X			
Routine coagulation tests (PT, aPTT and fibrinogen)^a^	X		X	X	X			
Additional clotting assays^b^			X	X	X			
Thromboelastographic assessment^a^			X	X	X			
Inform transfusion laboratory of need for FFP			X					
Randomisation and intervention – PCC or octaplasLG /FFP			X					
Time of intervention (start and stop)			X					
Weekly ICU assessment					X	X		
Thromboembolic AE/SAE				X	X	X	X	X
Transfusion AE/SAE				X	X	X	X	
Hospital re-admission since discharge								X
Quality of life – EQ-5D	X							X
90-Day survival status - end of study form (telephone or clinic visit)								X

*Abbreviations: AE* adverse events, *aPTT* activated partial thromboplastin time, *EQ-5D* EuroQol 5-dimension quality of life scale, *FBC* full blood count, *FFP* fresh frozen plasma, *ICU* intensive care unit, *PCC* prothrombin complex concentrate, *PT* prothrombin time, *SAE* serious adverse events

Clinical data that will be collected include age, sex, ethnicity, previous medical history, drug history, type of surgery and date/time of intervention. For those who have received intervention, daily and weekly (24 h and 7, 14, 21 and 30 days, or upon discharge, or death, whichever is first) assessments will be performed for amount of blood lost through the chest drains, blood components transfused (red blood cells, FFP, platelets and cryoprecipitate), any other haemostatic agents administered (such as recombinant factor VIIa, fibrinogen concentrate), total days in intensive care unit (level 3), high-dependency units (level 2), any organ failure (e.g., acute lung injury, acute respiratory distress syndrome, renal failure, liver failure), thrombosis (arterial and venous thrombosis), acute transfusion reaction, infections, duration of organ support (i.e., ventilatory support, cardiovascular support, and renal replacement therapy), and mortality. At 90 days or death, whichever is first, the following data will be collected: mortality, re-hospitalisation, thromboembolic event (arterial and venous), number of days alive and out of hospital since operation, and quality-of-life questionnaire.

### Statistics

#### Sample size calculation

Over a 15-month period, we expect 638 patients to be eligible. This would allow us to estimate a consent rate of 30% within a 95% confidence interval of ± 3.5%. Assuming that 30% of the eligible patients consent, we will have a sample of 191 patients on the basis of which to estimate the proportion of consented patients who bleed and are administered FFP/PCC. From the national and local cardiac audit data, the rate of FFP transfusion in the eligible study patients is just over 30%, so we have estimated that 30% of consented patients will go on to develop bleeding during surgery that requires FFP transfusion. A sample size of 191 would allow us to estimate a proportion of 30% within a 95% confidence interval of ± 6.5%. On the basis of the above 30% rate, around 57 patients would be randomised within 15 months, giving an expected final sample size of 50 patients completing the study after allowing for 10% drop-out or loss to follow-up. This sample would be analysed for assessment of the secondary endpoints. No formal interim analysis for efficacy is planned. Numbers recruited, eligibility and consent rates will be considered by the data and safety monitoring committee (DSMC). Safety analysis including reporting of adverse events will be undertaken biannually for review by the DSMC. Other interim analysis may be undertaken at the request of the DSMC. Tables will be prepared by the study statistician.

The primary analysis will use data from the eligible patient population (for consent rate estimation) and the consenting patients (for estimation of the percentage who are randomised and receive study treatment). The proportion of patients who agree to collection of their de-identified data for up to 24 h after surgery will be obtained to analyse the population of eligible patients who do not consent to enter the main trial. The intention-to-treat population will be used to analyse secondary endpoints relating to the delivery of the intervention, clinical outcome data and haemostatic capacity of patients. Full details of the statistical considerations are given in the study statistical analysis plan.

## Discussion

There has been no RCT that has compared the clinical efficacy and safety of PCC versus FFP in patients undergoing cardiac surgery who are bleeding and have not been taking a vitamin K antagonist in the peri-operative phase. Observational studies have suggested that PCC is safe in this setting; however, clinical equipoise and the lack of high-quality evidence mean that a large RCT is required to determine how PCC compares with FFP. Prior to such a trial, it is important to assess the feasibility of recruitment and different aspects of delivering the large trial, and this is the aim of this pragmatic, pilot RCT.

The pragmatic nature of the study means that the decision whether to administer the intervention will be based on ‘real-world’ practice rather than on a specific algorithm. One reason for choosing this approach is that it is vital that the results produced from the study are applicable to everyday practice in the future. Further, a recent phase III RCT in a cardiac surgery setting that compared fibrinogen concentrate with placebo highlighted some of the challenges with trials using complex algorithms to administer intervention [8]. Difficulties in implementing such algorithms during trials can result in a number of shortcomings, such as low proportion of patients being actually randomised, high rate of non-adherence to the study protocol, high proportion of patients being given the intervention when they did not fulfil the study criteria, and consequently greater costs incurred.

Furthermore, the pragmatic nature of the trial reflects real-world current practice and does not add pre-intervention tests that could delay the issuing of FFP or PCC in a clinical scenario that requires rapid action. The pilot study will collect pre-intervention clotting profile data, but these data will not be used as entry criteria to allow the intervention to take place. There is no current bedside test with 100% sensitivity and specificity to identify the need for blood products after cardiac surgery, and as such the trial reflects real-world practice and current clinical judgement. There is no set limit for the amount of blood loss to define bleeding, because although this is possible with a closed chest and chest drains in an intensive care unit, it is not possible to define in the operating room before chest closure when swabs and suction are being used.

Another important aspect of this pilot trial are the surveys with different experts across disciplines (e.g., cardiac surgeons, anaesthetists, intensivists, transfusion laboratory scientists) and patient and public groups to reach a consensus on the outcome measures for the large trial. In 2015 Benstoem and colleagues [9] performed a systematic review of the literature to identify the main outcomes that have been measured in cardiac surgery intervention trials in adults; in this review a total of 121 outcomes were identified, which were collapsed into 36 outcome domains. Using the results of the above review, in 2017 Benstoem and colleagues [10] performed an international three-round eDelphi exercise to reach a consensus on core outcome sets that should be measured and reported, at minimum, in clinical trials of cardiac surgery. Of the 36 outcome variables identified from the systematic review, the panel reached consensus on four core outcome sets, which were mortality, quality of life, hospitalisation and cerebrovascular complications*.* Currently in the United Kingdom a national database is used to collect clinical outcomes of patients who have undergone cardiac surgery, and of the 4 core outcome sets agreed in the Delphi consensus [10], quality of life is the only outcome that is not collected by the national database, and of the 36 outcome variables identified from the systematic review [9], a total of 7 variables are collected in the United Kingdom. In order to obtain patient and public opinion about the outcome measures for the large trial, we will conduct surveys with patients and UK healthcare professionals, using the results of the above Delphi survey and the outcomes measured by the national database. Further, we will also conduct interviews with patients and clinicians who have been involved with the study to explore understanding of, and experience with, the intervention delivered to get their input on how best to optimise recruitment of participants and how to improve adherence of the trial protocols. All of these efforts will allow for a more cost-effective and informative trial in the future.

## Trial status

Protocol V2.0, 27 November 2018. Start date of subject recruitment: 1 March 2019. Project recruitment completion date: 30 June 2020.

The study was peer-reviewed by three independent experts as part of the BHF funding application and underwent Barts Heart Centre independent peer review. The study protocol has been reviewed by the Barts Cardiovascular Clinical Trials Unit (CVCTU) Scientific Committee and the Blizard Institute, Medicines and Healthcare products Regulatory Agency (MHRA) and NHS research ethics committees.

CVCTU will oversee the management and conduct of the trial and will be responsible for pharmacovigilance and safety reporting, coordination of trial committees, statistical analysis and reporting, and database management and case report form (CRF) design. The study sponsor will be responsible for trial monitoring. When the research trial is complete, it is a sponsor requirement that the records be kept for a further 20 years in a secure, long-term storage facility as per the sponsor policy.

Data will be captured in REDCap, a web-based electronic database, for all study participants, and the database will be held on a secure server at Queen Mary University of London (QMUL). Participants eligible for the study will be given a screening number, and this number will be used to identify them throughout the study duration. The screening number will be identified on all electronic case report forms (eCRFs) and study documentation (e.g., questionnaires, laboratory reports, enrolment and dispensing logs). Only authorised users approved by the chief investigator (CI) will have access to the REDCap electronic database, and each user will be assigned specific user roles and rights. Sponsor representatives and CVCTU team members will have read-only access to the data. The study research nurse will be the primary person with delegated responsibility for data entry and CRF completion. The transfusion laboratory team will have access to the eCRF to complete randomisation. The CI will have overall responsibility for data captured in the eCRF and be able to review, lock and electronically sign the completed eCRFs.

### Supplementary information

**Additional file 1.** SPIRIT 2013 Checklist.

## Data Availability

The datasets used and/or analysed during the current study will be available from the CI on reasonable request. All data generated or analysed during this study will be included in future publications.

## Abbreviations

AE: Adverse events
aPTT: Activated partial thromboplastin time
CI: Chief investigator
CRF: Case report form
CVCTU: Cardiovascular Clinical Trials Unit
DSMC: Data and safety monitoring committee
EQ-5D: EuroQol 5-dimension quality of life scale
FBC: Full blood count
FFP: Fresh frozen plasma
ICU: Intensive care unit
IgA: Immunoglobulin A
MHRA: Medicines and Healthcare products Regulatory Agency
PCC: Prothrombin complex concentrate
PROPHESY: Trial of PCC Versus FFP in Patients Undergoing Heart Surgery
PT: Prothrombin time
QMUL: Queen Mary University of London
RCT: Randomised controlled trial
SAE: Serious adverse events
TSC: Trial steering committee
